# Genome-wide analysis of the R2R3-MYB gene family in *Spatholobus suberectus* and identification of its function in flavonoid biosynthesis

**DOI:** 10.3389/fpls.2023.1219019

**Published:** 2023-08-21

**Authors:** Shuangshuang Qin, Fan Wei, Ying Liang, Danfeng Tang, Quan Lin, Jianhua Miao, Kunhua Wei

**Affiliations:** ^1^ National Center for Traditional Chinese Medicine (TCM) Inheritance and Innovation, Guangxi Botanical Garden of Medicinal Plants, Nanning, China; ^2^ Guangxi Key Laboratory of Medicinal Resources Protection and Genetic Improvement, Guangxi Botanical Garden of Medicinal Plants, Nanning, China

**Keywords:** *Spatholobus suberectus* Dunn, R2R3-MYB gene family, genome-wide analysis, flavonoid biosynthesis, expression analyses

## Abstract

*Spatholobus suberectus* Dunn (*S. suberectus*), a plant species within the Leguminosae family, has a long history of use in traditional medicines. The dried stem of *S. suberectus* exhibits various pharmacological activities because it contains various flavonoids. Diverse functions in plants are associated with the R2R3-MYB gene family, including the biosynthesis of flavonoids. Nonetheless, its role remains unelucidated in *S. suberectus.* Therefore, the newly sequenced *S. suberectus* genome was utilized to conduct a systematic genome-wide analysis of the R2R3-MYB gene family. The resulting data identified 181 *R2R3-SsMYB* genes in total, which were then categorized by phylogenetic analysis into 35 subgroups. Among the *R2R3-SsMYB* genes, 174 were mapped to 9 different chromosomes, and 7 genes were not located on any chromosome. Moreover, similarity in terms of exon-intron structures and motifs was exhibited by most genes in the same subgroup. The expansion of the gene family was primarily driven by segmental duplication events, as demonstrated by collinearity analysis. Notably, most of the duplicated genes underwent purifying selection, which was depicted through the Ka/Ks analysis. In this study, 22 *R2R3-SsMYB* genes were shown to strongly influence the level of flavonoids. The elevated expression level of these genes was depicted in the tissues with flavonoid accumulation in contrast with other tissues through qRT-PCR data. The resulting data elucidate the structural and functional elements of *R2R3-SsMYB* genes and present genes that could potentially be utilized for enhancing flavonoid biosynthesis in *S. suberectus*.

## Introduction

Constituting one of the largest and well-studied transcription factor (TF) gene families in plants, the MYB TFs, are an extensive family widely involved in various types of plant growth and development (Riechmann et al., 2000; Zhao et al., 2017; Yuan et al., 2021). The presence of MYB DNA binding domain (DBD) is a defining feature of these genes. This domain comprises around 50-55 amino acids, is highly conserved, and adopts a helix-turn-helix (HTH) structure (Ogata et al., 1996). Furthermore, these TFs can be categorized as per the number of MYB domains into four types:1R-MYB (comprising a single or a partial MYB repeat), R2R3-MYB (2R-MYB), R1R2R3-MYB (3R-MYB) and 4R-MYB (comprising four R1/R2 repeats) (Dubos et al., 2010). The R2R3-MYB subfamily is the largest among these, found in various plant species (Sundararaman et al., 2015; Pu et al., 2020). Currently, the identification of R2R3-MYB transcription factors at the genomic level has been carried out through the sequencing of various plants, such as 138 *R2R3-AtMYBs* among 197 *AtMYB* transcription factors in *Arabidopsis thaliana* (Katiyar et al., 2012), 106 *R2R3-PaMYBs* among 155 *PaMYB* transcription factors in *Petunia axillaris* (Chen et al., 2021), and 244 *R2R3*-*GmMYB* among 252 *GmMYB* transcription factors in *Glycine max* (Du et al., 2012).

A majority of *R2R3-MYB* genes are largely conserved in different species, and the similarity of their sequence allows them to be categorized into the same subgroups. Nevertheless, interspecies variations still exist. As demonstrated by a plant study that exhibited extensive evolutionary expansion of this gene family by comparatively analyzing these genes across various species (Ito, 2005; Zhuang et al., 2021). The *R2R3-MYB* genes expansion is involved in a variety of developmental and growth processes in plants as well as disease resistance, hormone signal transduction, and biotic and abiotic stress (Aoyagi et al., 2014; Anwar et al., 2019; Wang et al., 2021; Zhuang et al., 2021; Yang et al., 2022). Increasingly, the research data of various plant species have implicated R2R3-MYB TFs in the modulation of secondary metabolism (particularly flavonoid biosynthesis and metabolism). In *Erigeron breviscapus*, *MYBP1* acts as a positive regulator and is linked to the regulation of flavonoid accumulation. It activates the transcription of flavonoid-associated genes by directly binding to their promoters (Zhao et al., 2022). *EsMYB9*, a subfamily of R2R3-MYB TFs, regulates the flavonoid biosynthesis pathway in *Epimedium sagittatum* by activating the expression of the chalcone synthase promoter (Huang et al., 2017).

Numerous studies on crop and horticultural plants concerning the members of the R2R3-MYB gene family have added to the existing data regarding their functions, evolutionary history, and transcriptional regulatory mechanism. Nonetheless, their functions in *Spatholobus suberectus* Dunn (*S*. *suberectus*), a traditional Chinese medicinal herb known as jixueteng, are not well understood. The dried stem of this plant depicts diverse pharmacological activities, and the primary bioactive constituents were determined to be flavonoids (Wang et al., 2017; Song et al., 2022). Catechin, the flavonoid with the highest content, can promote the proliferative capacity of the hematopoietic progenitor cells. Additionally, genistein, isoliquiritigenin, and formononetin have been demonstrated to have efficacy in cancer preventive or therapeutic strategies (Wang et al., 2008; Wang, 2011; Peng et al., 2016). At present, these four flavonoid biosynthetic pathways have been well elucidated in *S. suberectus*, and over 70% of genes involved in flavonoid biosynthesis had MYB binding sites in their promoter regions (Qin et al., 2020). This result verified the role of MYB TFs in regulating intermediates in the flavonoid biosynthesis pathway. However, the members of the *R2R3-SsMYB* gene family concerning their modulation of flavonoid biosynthesis remain to be investigated.

As described in our previous study, the genome of *S. suberectus* has been sequenced (Qin et al., 2019), facilitating a genome-wide analysis and identification of the functions of genes in the *R2R3-SsMYB* family. In the present study, we conducted a genome-wide analysis of the *R2R3-SsMYB* family, including sequence features, phylogenetic relationships, gene structure, motif recognition, collinearity, and chromosomal location. Candidate *R2R3-SsMYB* genes associated with flavonoid biosynthesis in the correlation analysis were identified and assessed by qRT-PCR. Our study not only serves as a comprehensive analysis of various characteristics of the *R2R3-SsMYB* family but also provides valuable insights for further functional assessment of the genes involved in flavonoid biosynthesis.

## Methods

### Identification of *Spatholobus suberectus* R2R3-MYB genes

The Hidden Markov Model (HMM) profile of the MYB DNA-binding domain with accession number (PF00249) was accessed at the Pfam database (http://pfam.xfam.org/) (Finn et al., 2016). This profile was utilized as a query for an HMM search in the *S. suberectus* genome with default parameters using HMMER version 3.0 (Finn et al., 2011) for the identification of MYB genes. NCBI’s Conserved Domains Database (CDD) (http://www.ncbi.nlm.nih.gov/Structure/cdd/wrpsb.cgi) and the database Simple Modular Architecture Research Tool (SMART) (http://smart.embl-heidelberg.de/) were used to confirm the acquired R2R3-MYB protein sequences in *S. suberectus.*

### Sequence analysis and structural characterization of *R2R3-SsMYB* genes

All *R2R3-SsMYB* genes were imported into the ProtParam tool (http://web.expasy.org/protparam/) to assess the isoelectric point, molecular weight, number of amino acids, and aliphatic and instability indices. The structures of all *R2R3-SsMYB* genes were visualized with the TBtools version 1.045 software using the genomic sequences and coding regions of the *R2R3-SsMYB* genes, with the lengths and numbers of the exons and introns included (Chen et al., 2020). The conserved motifs of the R2R3-MYB protein sequences were using the motif-based sequence analysis tool MEME Suite (https://meme-suite.org/meme/tools/meme) (Bailey et al., 2009). The analysis was set to identify a maximum of 20 motifs with an optimum motif width range of 6 to 100 amino acids (Zhuang et al., 2021).

### Phylogenetic analysis of *R2R3-SsMYB* genes

Phylogenetic trees were generated utilizing the R2R3-MYB protein sequences of *S. suberectus* and *A. thaliana*. We performed multiple sequence alignment using MAFFT version 7.427 with the default parameters for better alignment speed and accuracy. A maximum likelihood (ML) phylogenetic tree was constructed using the software Molecular Evolutionary Genetics Analysis (MEGA) version 7.0 and the following parameters: Poisson model, partial deletion, and 1000 bootstrap replicates. Visualization of the tree was executed through the software FastTree (Price et al., 2009). Furthermore, similar methods were employed for the establishment of a separate phylogenetic tree with all the R2R3-SsMYB protein sequences. The Tbtools version 1.045 (Chen et al., 2020) was utilized to create a visual representation combining the gene structures, phylogenetic tree, and conserved motifs of the R2R3-SsMYB protein sequences.

### Genomic localization, collinearity analysis, and gene duplication of *R2R3-SsMYB* genes

The physical positions of identified *R2R3-SsMYB* genes were mapped to nine chromosomes of *S. suberectus* using MapGene2Chrom (MG2C) (http://mg2c.iask.in/mg2c_v2.0/), a tool for quickly drawing physical gene maps in SVG format based on the input data (Chao et al., 2021). The determination and visualization of the collinearity of the interspecific and intraspecific genes were conducted through the Multiple Collinearity Scan toolkit (MCSscanX) and the circos multiple synteny plot, respectively. The parameters of MCSscanX were as follows: gap_penalty: −1, *E*-value: 1e-10 (Chao et al., 2021). The determination and visualization of the collinearity of the interspecific and intraspecific genes were conducted through the Multiple Collinearity Scan toolkit (MCSscanX) and the circos multiple synteny plot, respectively (Wang et al., 2012). To estimate duplication events, the nonsynonymous (K_a_) and synonymous (K_s_) substitution rates and evolutionary constraint (K_a_/K_s_) between the duplicated pairs of *R2R3-SsMYB* genes were calculated using KaKs_Calculator 2.0 (Wang et al., 2010). Circos version 0.69 was used to graphically present the synteny blocks of the orthologous *R2R3-MYB* genes between *S. suberectus* and *A. thaliana*, *S. suberectus* and *G. max* (Krzywinski et al., 2009).

### Expression analyses by RNA−seq and correlation analysis of *R2R3-SsMYB* genes

The expression pattern of *R2R3-SsMYB* genes in the root, stem, and other tissues were examined by retrieving the transcriptomic data of the putative *R2R3-SsMYB* genes in the various tissues from prior research that utilized these gene IDs as the queries (Qin et al., 2020). The abundance of *R2R3-SsMYB* transcripts was presented in the format of fragments per kilobase (of exon model) per million mapped reads (FPKM). To cluster genes with the same or similar expression, we conducted hierarchical clustering on the log_2_^(FPKM+1)^ from the RNA-seq data using Cluster version 3.0 and visualized the resulting data with Java TreeView (Zhuang et al., 2021). In order to further investigate *R2R3-SsMYB* related to flavonoid components, the contents of flavonoid, catechin, genistein, isoliquiritigenin, and formononetin in the different tissues were obtained from previous research (Qin et al., 2020). The relationship between *R2R3-SsMYB* genes and flavonoid content was analyzed by Spearman rank correlation analysis in R version 3.6.2, and *p* < 0.05 was considered statistically significant.

### Expression analyses of *R2R3-SsMYB* genes by qRT-PCR

The Guangxi Botanical Garden of Medicinal Plants was utilized for the cultivation of the *S. suberectus* plants with samples from leaves, stems, roots, fruits, and flowers acquired from 12-year-old plants. Each plant tissue was sampled in triplicate. The plant samples were exposed to liquid nitrogen for freezing with subsequent storage at −80°C for RNA extraction. Extraction of the total RNA utilized the FastPure Universal Plant Total RNA Isolation Kit (Vazyme, China). Gel electrophoresis and NanoDrop 2000 spectrophotometer (Thermo Scientific, United States) were employed for assessing the quality and concentration of the RNA samples. HiScript ® III RT SuperMix for qPCR (Vazyme, China) was designed for the synthesis of cDNA. The amplification of 20μl final volume involved the StepOne real-time PCR system (Applied Biosystems, United States) as well as the ChamQTM Universal SYBR® qPCR Master Mix (Vazyme, China). The qRT-PCR reactions involved three biological replicates, and *18S* was chosen as an endogenous reference gene (Ferradas et al., 2016). The amplification specificity was examined by melting curve analysis, and the degree of expression of the gene was calculated using the 2^−ΔΔCT^method. To test for significant differences between samples, we employed Duncan’s multiple range test (SPSS version 17.0). Primer version 5.0 was utilized for designing the specific *R2R3-SsMYB* primers (**Supplementary Table 1**).

### Statistical analysis

The three biological replicates were utilized in the experiments. R software was utilized for statistical significance (Student’s t-tests) with the significance level set at *p* < 0.05.

## Results

### Identification and characterization of *Spatholobus suberectus R2R3-MYB* family genes

The assessment of the plant genome resulted in identifying 272 candidate genes that coded for MYB domain-containing proteins. All candidate genes were further categorized into four major subfamilies, which included 81 *1R-SsMYBs*, 181 *R2R3-SsMYBs*, 9 *R1R2R3-SsMYBs*, and 1 *4R-SsMYB*. Among *R2R3-SsMYB* genes, 174 *R2R3-SsMYB* genes were mapped to 9 chromosomes and renamed from *SsMYB1* to *SsMYB174* according to their location on the chromosomes. In addition, 7 *R2R3-SsMYB* genes were not located on any chromosome.

Involving 123 to 554 amino acids, the R2R3-SsMYB proteins ranged in molecular weight values and theoretical isoelectric point from 13.91 (*SsMYB174*) to 60.88 (*SsMYB33*) kDa and 4.61 (*SsMYB40*) to 9.96 (*SsMYB57*), respectively. Additionally, they were subjected to subcellular localization, revealing that 147 of 181 (around 81%) R2R3-SsMYB proteins were present in the nucleus (**Supplementary Table 2**).

### Phylogenetic analysis and categorization of *R2R3-MYB* genes in *Spatholobus suberectus*

**Figure 1:**
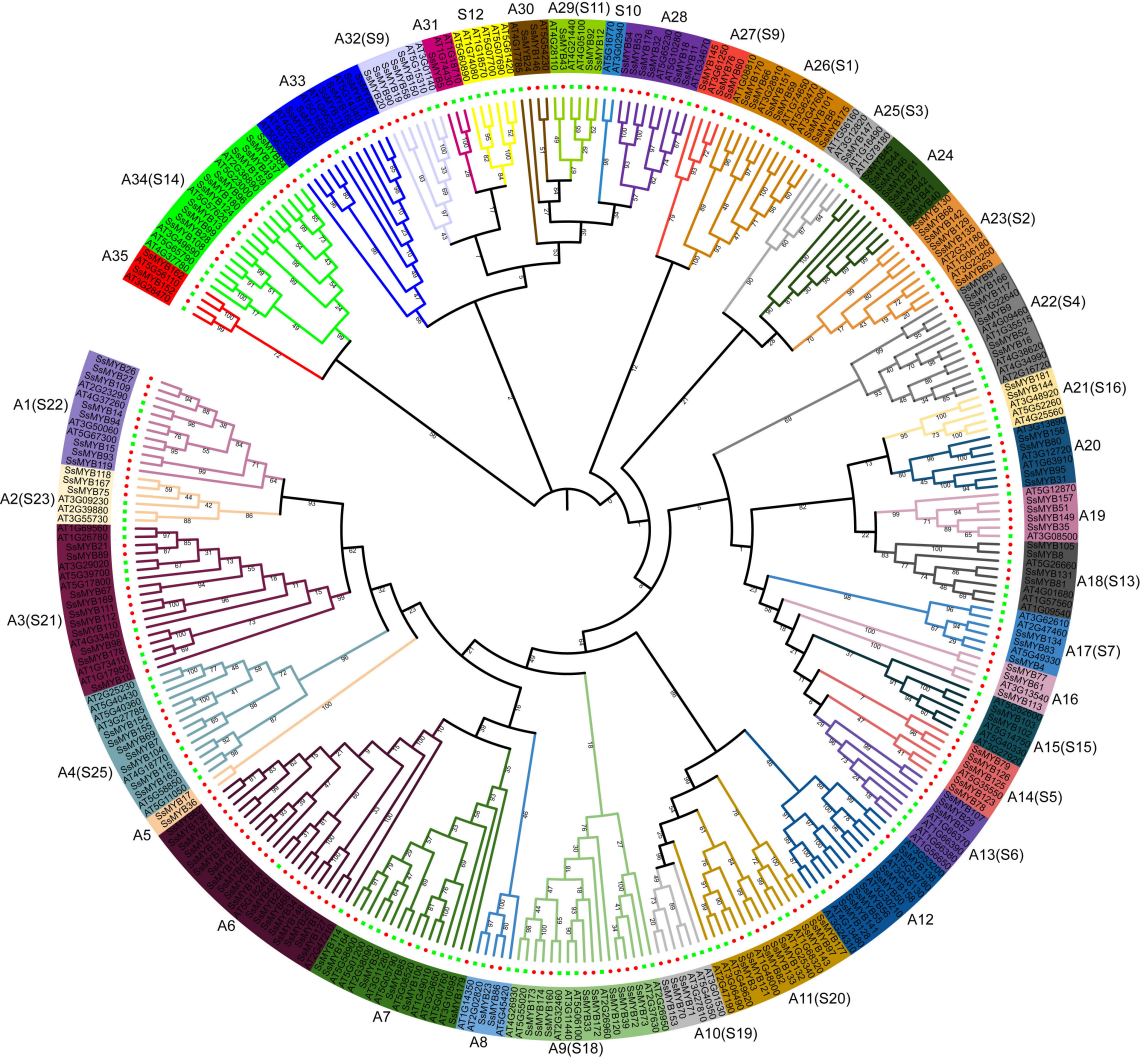
Comparison of the phylogenetic tree of R2R3-MYB proteins between *Spatholobus suberectus* (*S. suberectus*) and *Arabidopsis thaliana* (*A. thaliana*). Distinct separation of subfamilies of R2R3-MYBs into clades (denoted by various colors). Generation of 35 subgroups of the genes of the *R2R3-SsMYB* family (designated as A1–A35).

The functions associated with the *R2R3-SsMYB* genes and their evolutionary history were examined in an ML tree established through FastTree. This tree comprised 181 *R2R3-SsMYB* and 138 *R2R3-AtMYB* genes (**Figure 1**). The 181 *R2R3-SsMYB* genes were categorized into 35 subgroups (A1–A35), with 22 of the groups (comprising 117 *R2R3-SsMYB* genes) exhibiting congruence with the *R2R3-AtMYB* proteins phylogenetic tree that was established previously. Additionally, 13 specific subgroups in *S. suberectus* did not cluster with *A. thaliana*. Furthermore, A5 and A24 subgroups just included *R2R3-SsMYB* genes but no *R2R3-AtMYB*, indicative of the occurrence of these genes in *S. suberectus* during the evolutionary process. S10 and S12 subgroups only contained *R2R3-AtMYB* genes with no *R2R3-SsMYB* genes, revealing that some evolutionary alterations were present in the genome, and the *R2R3-MYB* genes could have been acquired in *A. thaliana* or lost in *S. suberectus* during evolution. The gain and loss of species-specific *R2R3-MYB* genes could have resulted in functional divergence.

### Conserved gene structure and motif composition of *R2R3-SsMYBs*

**Figure 2:**
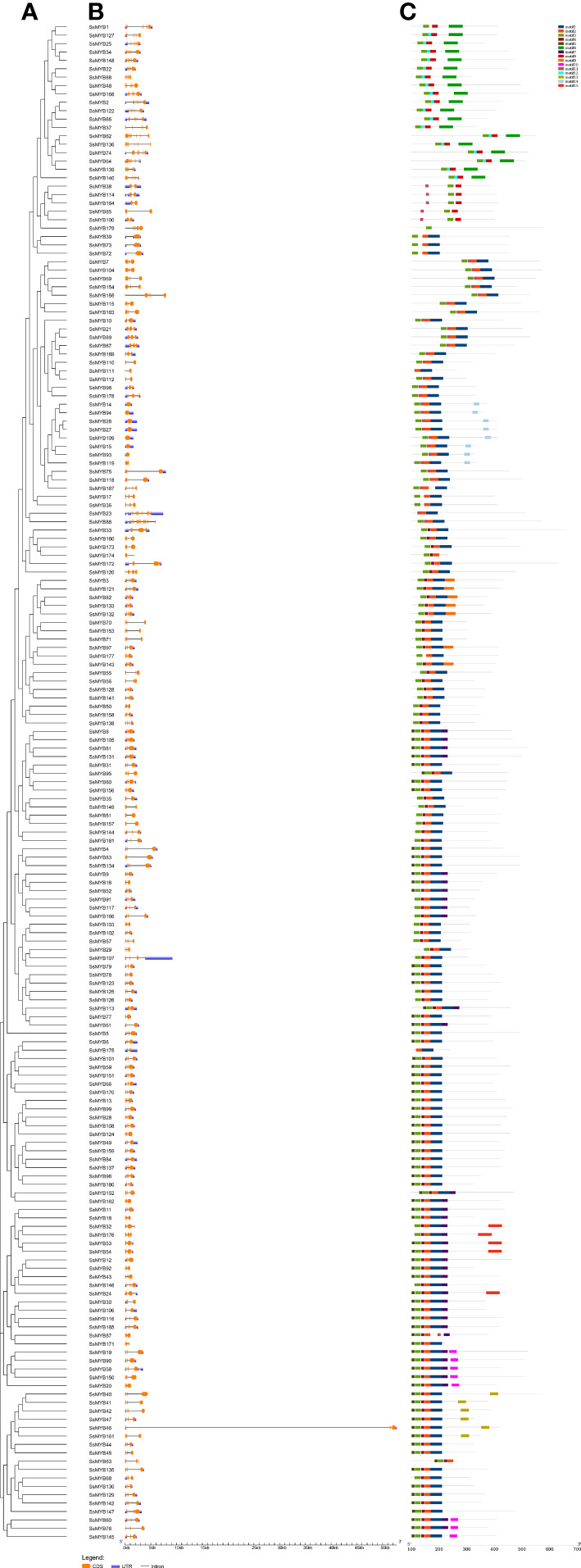
Analysis of gene structure and conserved motifs depending on the phylogenetic relationships in *R2R3-SsMYB* genes. **(A)** Phylogenetic tree established utilizing 181 *R2R3-SsMYB* proteins with the ML method. **(B)** Exon/intron structure analysis of *R2R3-SsMYB* genes. Respective black lines, as well as orange and blue boxes, denote introns, exons, and untranslated regions (UTRs). **(C)** Conserved motifs of *R2R3-SsMYB* genes elucidated by MEME Suite (represented by the various colored boxes). The scale bar of each *R2R3-SsMYB* gene is shown at the bottom.

Structural analysis of genes provided information on gene function and evolution. The pattern of the exon-intron structure of *R2R3-SsMYB* genes was examined to gain insight into their structural diversity and the composition of their motif (**Figures 2A, B**). The resulting data indicated a total of 181 *R2R3-SsMYB* genes with exons varying from 1 to 11, constituting 98.3% of the total *R2R3-SsMYB* genes. The majority (105 of 181) of the *R2R3-SsMYB* genes had typical splicing (two introns and three exons), where a single exon was depicted in 8 *R2R3-SsMYB* genes that had no intron, such as *SsMYB14*, *SsMYB15*, *SsMYB26*, *SsMYB27*, *SsMYB39*, *SsMYB72*, *SsMYB73*, and *SsMYB109*. Characteristics of gene structure, including the number of introns, were included in the phylogenetic analysis of the *R2R3-SsMYB* family. Genes within the same subgroup exhibited similarity in exon–intron structure due to the fully conserved position(s) of the intron(s). For example, *SsMYB38*, *SsMYB85*, *SsMYB100, SsMYB114*, and *SsMYB164* in subgroup A7 each contained two exons; *SsMYB8*, *SsMYB105*, *SsMYB81*, and *SsMYB131* in subgroup A18 each contained three exons; and *SsMYB1*, *SsMYB25*, *SsMYB34, SsMYB127*, and *SsMYB148* in subgroup A6 each contained six exons (**Supplementary Table 2**).

The 15 conserved motifs of all the R2R3-SsMYB proteins were predicted to further reveal the diversification of *S. suberectus* (**Figure 2C**, **Supplementary Table 3**). Motifs 1-5 were commonly found together in most of these proteins. Motif 3 was present in most genes, with the exception of *SsMYB23*, *SsMYB111*, and *SsMYB175*. In general, the composition of structural motifs varied among different subgroups but exhibited similarity within the same subgroup. Motif 13 was found only in *SsMYB40*, *SsMYB41*, *SsMYB42*, *SsMYB46*, *SsMYB47*, and *SsMYB161*, and these genes were clustered in subgroup A24. Motif 14 was found only in *SsMYB14*, *SsMYB15*, *SsMYB26*, *SsMYB27*, *SsMYB93, SsMYB94*, *SsMYB109*, and *SsMYB119*, and these genes were clustered in subgroup A1 (**Figure 2C**, **Supplementary Table 2**). The conserved motifs observed in specific subgroups suggest that R2R3-SsMYB proteins within the same subgroup, sharing these motifs, may perform similar functions, as evidenced by the results of the phylogenetic analysis.

### Chromosomal location of *R2R3-MYB* genes in *Spatholobus suberectus*

**Figure 3:**
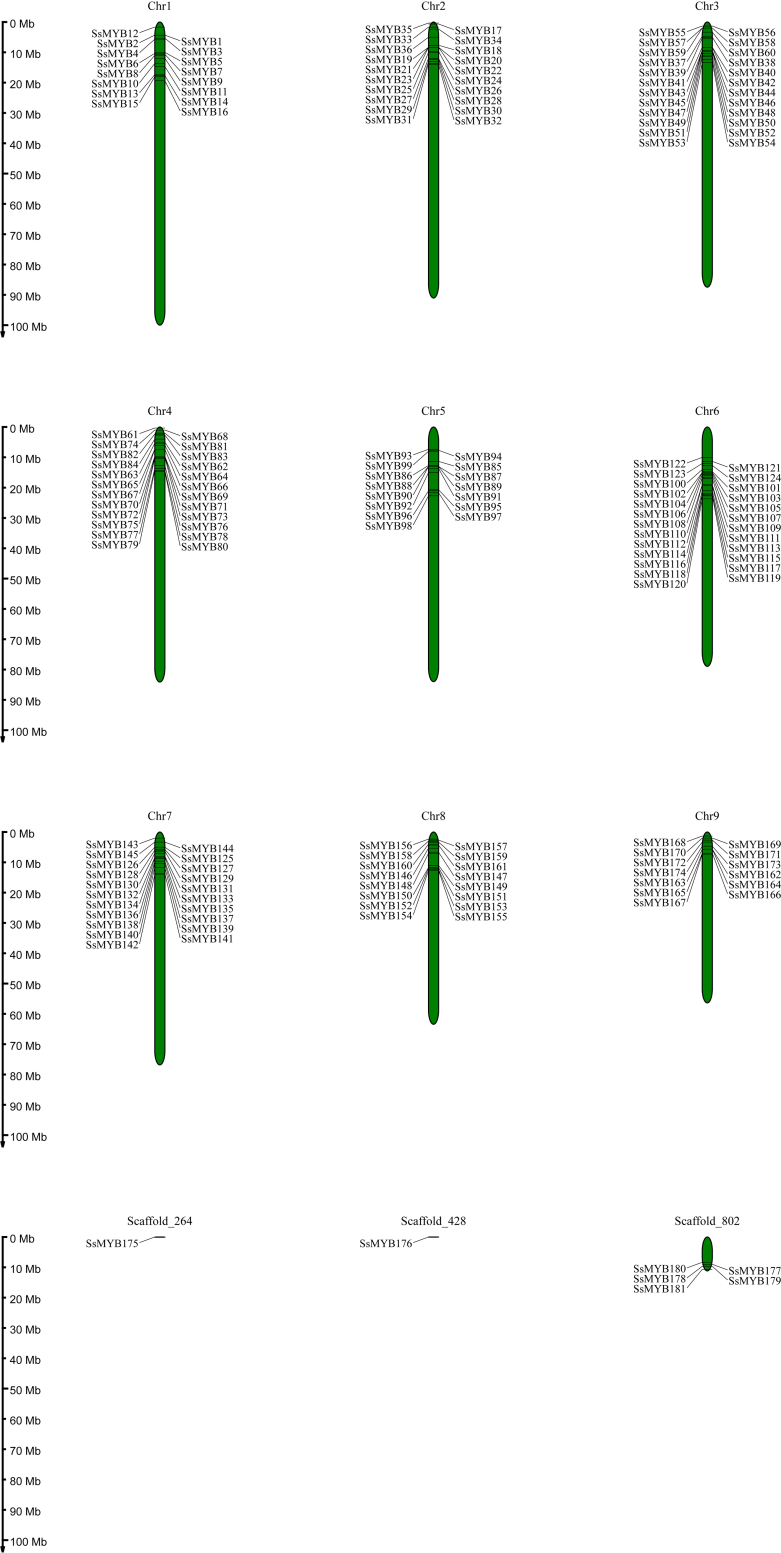
Chromosomal locations of *S. suberectus R2R3-MYB* genes. 174 *R2R3-SsMYB* genes mapped on 9 chromosomes, and the other 7 *R2R3-SsMYB* genes belonged to unassembled scaffolds. Chromosomal locations of *R2R3-SsMYB* genes were mapped based on the *S. suberectus* genome. The chromosome number is indicated at the top of each chromosome.

The *R2R3-SsMYB* gene sequences were utilized to determine their chromosomal locations. The resulting data depicted that this distribution was uneven, with 174 out of 181 *R2R3-SsMYB* genes present on 9 chromosomes: 16 *R2R3-SsMYB* genes were present on chromosomes 1 and 8; 20 on the 2^nd^; 24 on the 3^rd^ and 4^th^; 15 on the 5^th^; 25 on 6^th^; 21 on the 7^th^; 13 on the 9^th^. In terms of genes, the 6^th^ chromosome (25) was at the top, with the 3^rd^ and 4^th^ (24) following after, with the least number noted for the 9^th^ (13). Additionally, 7 *R2R3-SsMYB* genes were located on the scaffold, while most of the remaining genes were present at the ends of the chromosomes (**Figure 3**).

### Duplication events of *Spatholobus suberectus* R2R3-MYB genes

**Figure 4:**
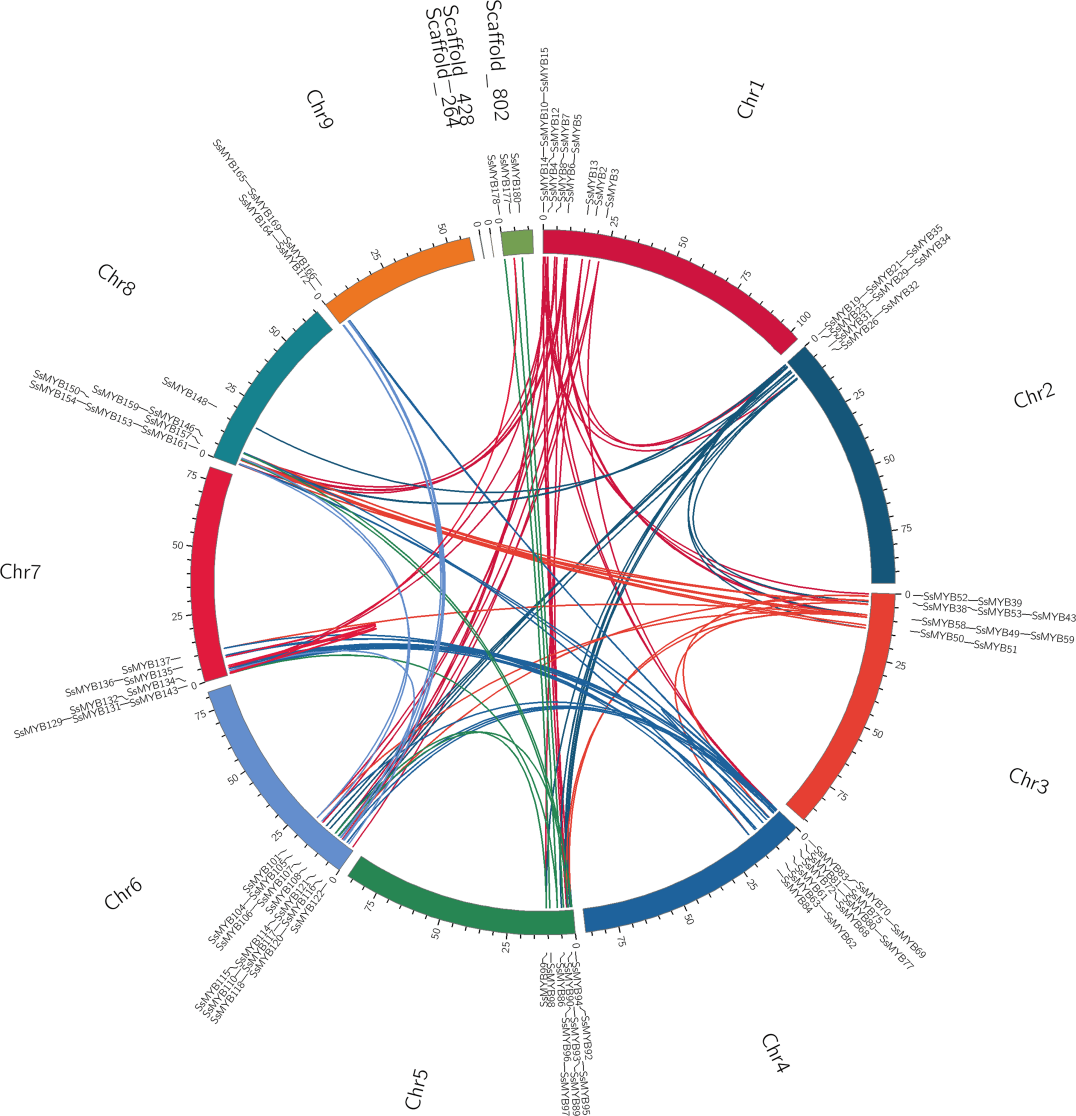
Collinearity analysis of the *R2R3-MYB* gene family in *S. suberectus.* All the synteny *R2R3-SsMYB* gene pairs were presented by curved lines and set as the same color.

Gene duplication, especially segmental and tandem duplication events, is a vital driver of the evolution and diversification of gene families. Establishing the collinearity of the *R2R3-MYB* genes in *S. suberectus* facilitated the assessment of their potential associations and duplication events (**Figure 4**). The current research determined that 100 duplicated gene pairs of *R2R3-SsMYB* existed in the *S. suberectus* genome and were primarily categorized as segmental duplication. *R2R3-SsMYB* gene pairs indicated as segmental duplications numbered 99, while only one pair of *R2R3-SsMYB* genes (*SsMYB55*/*SsMYB56*) were found as tandem repeats in *S. suberectus*. To evaluate the selection of the duplicated *R2R3-SsMYB* gene pairs, the non-synonymous to synonymous substitution ratios (Ka/Ks) were quantified by assessing the gene duplications by means of whole genome analysis. In the present study, the Ka/Ks ratios of all the *R2R3-SsMYB* gene duplicated pairs were lower than one, which suggests that these genes were subjected to purifying selection. This indicates that these duplicated genes are important for maintaining the functions of the *R2R3-SsMYB* family in *S. suberectus* (**Supplementary Table 4**).

**Figure 5:**
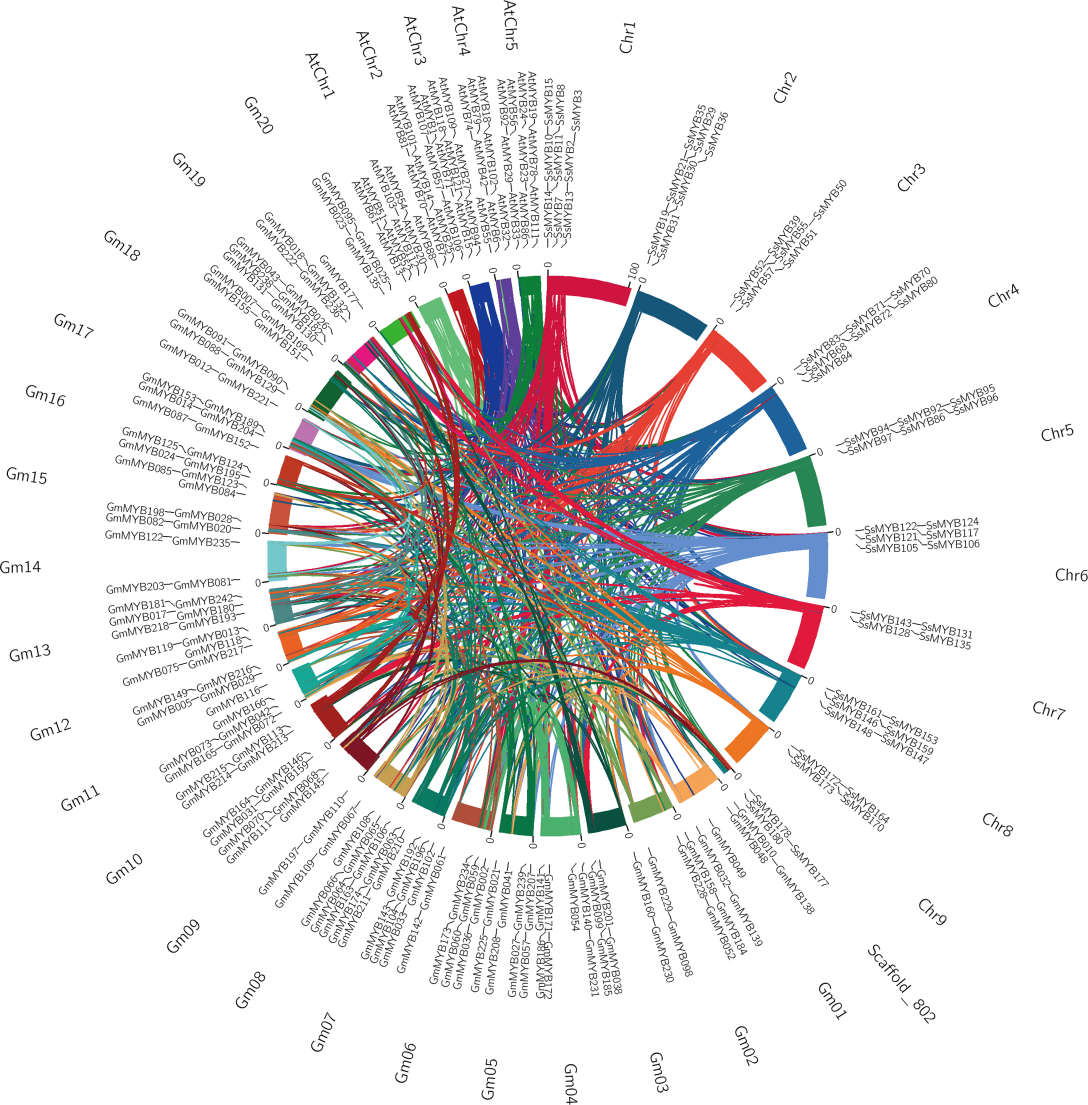
Collinearity analysis of R2R3-MYB genes between *S. suberectus* and two representative plant species [*A. thaliana* and *G. max*]. Presentation of gene pairs and syntenic R2R3-MYB gene pairs by curved lines and set as the same color.

To shed light on the *R2R3-SsMYB* family concerning its evolutionary history, *S. suberectus* and two other representative species, particularly, *A. thaliana* and *Glycine max* were analyzed comparatively through two orthologous analyses (**Figure 5**). There were 73 orthologs between *S. suberectus* and *A. thaliana* and 336 orthologs between *S. suberectus* and *G. max* (**Supplementary Tables 5, 6**). Previous studies had reported that *G. max* contains 20 chromosomes with a genome size of 950 Mb, while *A. thaliana* contains only five chromosomes with a genome size of 125 Mb (The Arabidopsis Genome Initiative, 2000; Schmutz et al., 2010). The higher chromosomal number and increased genomic size led to increased orthologous events in *SsMYB-GmMYB* in contrast with *SsMYB-AtMYB*.

### Determination of the upstream regulatory *R2R3-SsMYB* genes of flavonoid biosynthesis

**Figure 6:**
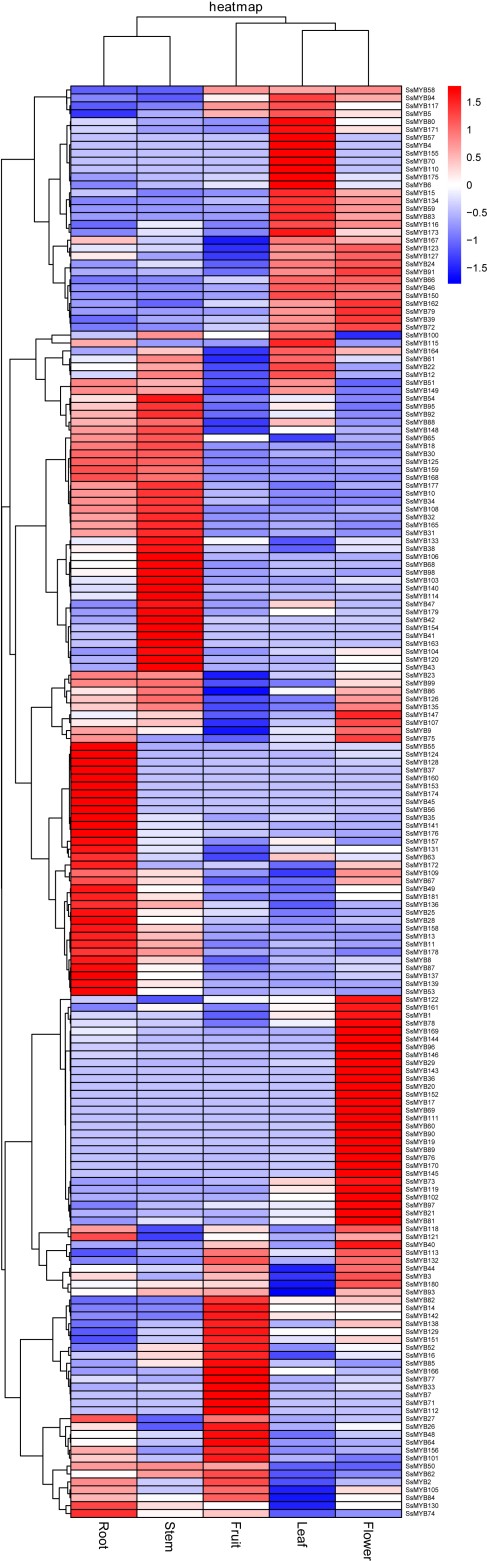
Expression levels of *R2R3-SsMYB* genes in roots, stems, leaves, flowers, and fruits using RNA-seq.

The *R2R3-SsMYB* genes were assessed among root, stem, and other tissues concerning their expression. The data procured previously were utilized to assess the expression levels of *R2R3-SsMYB* genes in the various tissues (**Figure 6**). Transcriptome analysis showed that the members of the *R2R3-SsMYB* family were differentially expressed in diverse tissues (**Supplementary Table 7**). Many *R2R3-SsMYB* transcription factors were expressed specifically in the stem, such as *SsMYB38*, *SsMYB47*, *SsMYB95*, *SsMYB106*, *SsMYB114*, and *SsMYB179*. The expression level of *SsMYB106* in the stem was 30.19 times higher than that in the leaf and 31.57 times that in the flower.

**Figure 7:**
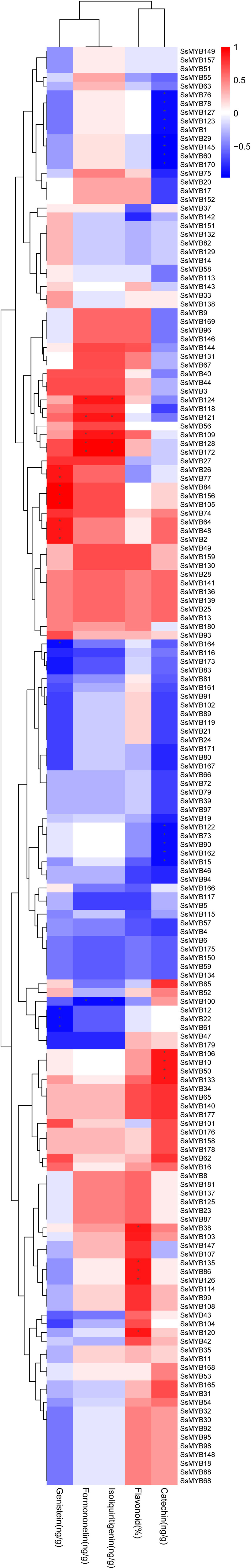
Correlation analysis between *R2R3-SsMYB* genes and flavonoid content was analyzed by Spearman rank correlation analysis in R (v3.6.2), and *p* < 0.05 (*) was considered statistically significant.

Flavonoid contents in percentage vary across the tissues (root, stem, leaf, flower, and fruit). The contents of flavonoid and catechin were the highest in the stem, the content of genistein was the highest in the fruit, and the contents of isoliquiritigenin and formononetin were the highest in the root (Qin et al., 2020). To further identify the upstream regulatory *R2R3-SsMYB* genes of flavonoid biosynthesis, correlation analysis was conducted with previous RNA-Seq and content data. *R2R3-SsMYB* genes remarkably linked to flavonoid, catechin, genistein, isoliquiritigenin, and formononetin contents were labeled (**Figure 7**). In detail, *SsMYB38*, *SsMYB86*, *SsMYB120*, *SsMYB126*, and *SsMYB135* were considerably linked to the flavonoid concentration. *SsMYB10*, *SsMYB50*, *SsMYB106*, and *SsMYB133* were strongly associated with catechin levels. *SsMYB2*, *SsMYB26*, *SsMYB48*, *SsMYB64*, *SsMYB77*, *SsMYB84*, *SsMYB105*, and *SsMYB156* were markedly correlated with genistein content. Furthermore, *SsMYB109*, *SsMYB121*, *SsMYB124*, *SsMYB128*, and *SsMYB172* were considerably linked to isoliquiritigenin and formononetin.

### Expression analyses of *R2R3-SsMYB* genes by qRT−PCR analysis

**Figure 8:**
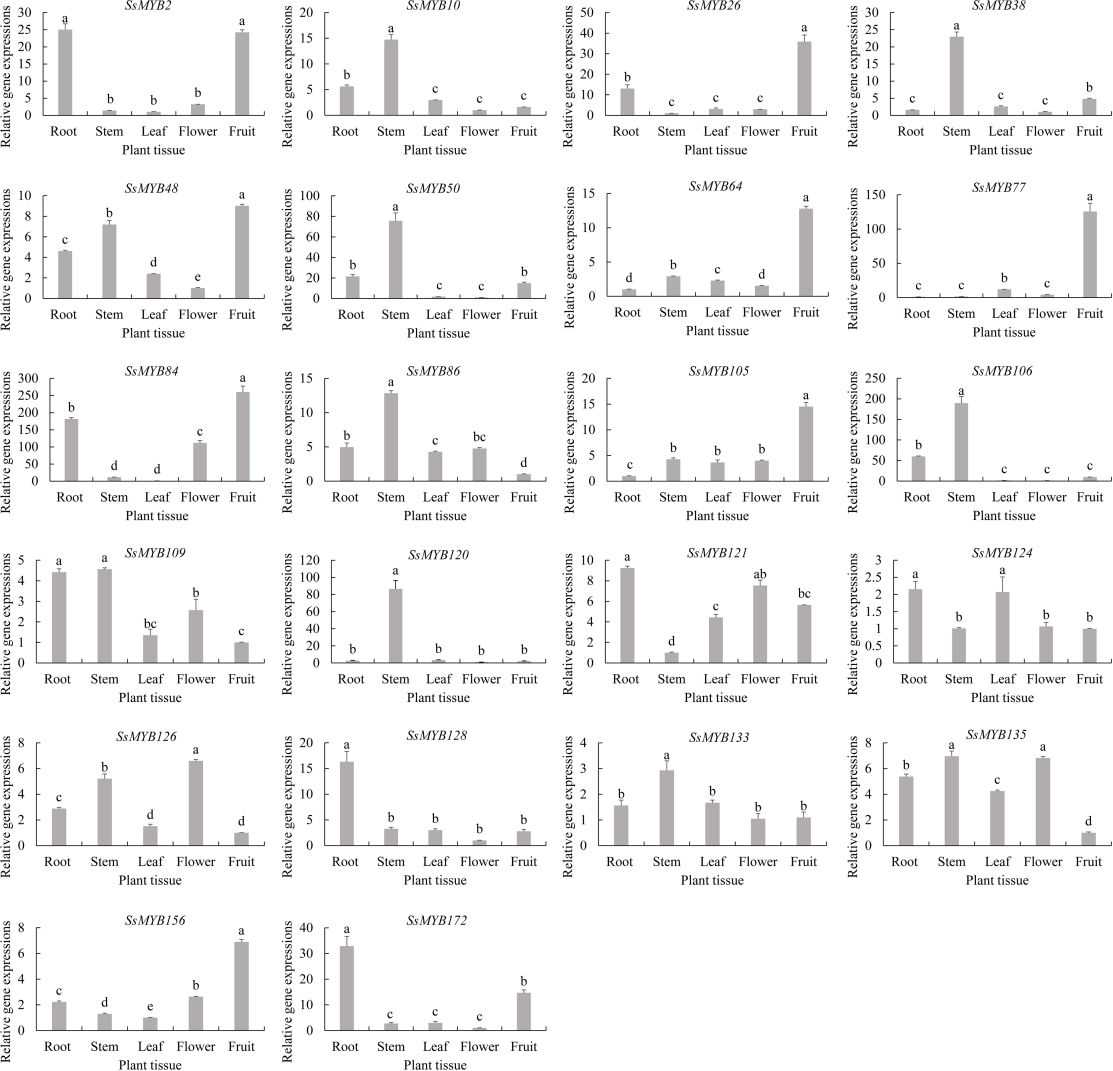
Expression analysis of 22 selected candidate *R2R3-SsMYB* genes in diverse tissues of *S. suberectus*. Indication of mean ± SE of three independent replicates through error bars. Various lowercase letters (a, b, c, d, and e) vary significantly (*p* < 0.05).

The pattern of *R2R3-SsMYB* genes regarding their expression in flavonoid biosynthesis was examined by assessing the candidate *R2R3-SsMYB* genes in five tissues of *S. suberectus* (**Figure 8**). Almost every *R2R3-SsMYB* gene in *S. suberectus* which was significantly correlated with flavonoid and catechin concentration, such as *SsMYB10*, *SsMYB38*, *SsMYB50*, *SsMYB86*, *SsMYB106, SsMYB120*, *SsMYB133* and *SsMYB135*, were specifically highly expressed in the stem.

*SsMYB2*, *SsMYB26*, *SsMYB48*, *SsMYB64*, *SsMYB77, SsMYB84*, *SsMYB105* and *SsMYB156*, which were significantly correlated with genistein content, were highly expressed, specifically in the fruit of *S. suberectus.* Most genes significantly correlated with the isoliquiritigenin and formononetin were specifically highly expressed in the root of *S. suberectus*, such as *SsMYB121*, *SsMYB128*, and *SsMYB172.* These tissues vary in terms of the proportion of flavonoids found in them which is likely affected by the varying expression patterns of the relevant genes. The constant and high expression of flavonoid biosynthesis-linked *R2R3-SsMYB* genes is the most probable cause of the increased expression of flavonoids in *S. suberectus* tissues.

## Discussion

### Identification and phylogenetics of *R2R3-MYB* genes in *Spatholobus suberectus*

Denoted as the largest subfamily among MYB TFs, the R2R3-MYB group is involved in various aspects of the secondary metabolism in plants (Anwar et al., 2019; Yang et al., 2022). Many *R2R3-MYB* genes have been identified in various species of plants. The data indicated that 138, 106, and 244 *R2R3-MYB* genes are present in *A. thaliana*, *P. axillaris*, and *G. max*, respectively (Du et al., 2012; Katiyar et al., 2012; Chen et al., 2021). The current research noted that 181 *R2R3-SsMYB* genes were detected in the *S. suberectus* genome. *R2R3-SsMYBs* accounted for 66.5% of the identified *SsMYB* gene family, which bears similarity to the proportion of *R2R3-MYB* genes in *P. axillaris* (68.3%) (Chen et al., 2021). In this study, *R2R3-SsMYB* genes were categorized into 35 subgroups. Although the number of observed subgroups was higher than that of *Pogostemon cablin* (31 subgroups; Zeng et al., 2023), it was lower than those of *Fragaria* × *ananassa* (37 subgroups; Liu et al., 2021) and *Musa acuminata* (42 subgroups; Pucker et al., 2020). The evolutionary origins and conserved functions of *R2R3-SsMYB* members are thought to be shared within the specific clades. As a result, based on the *R2R3-AtMYBs* functional clades, the putative functions of *S. suberectus* R2R3-MYB proteins can be speculated. Phylogenetic analyses and evolutionary relationships of the *R2R3-SsMYB* genes have been systematically studied among different species.

### Gene structure and protein motif analysis of *R2R3-MYB* genes in *Spatholobus suberectus*

The pattern of gene structure is a useful tool for studying the evolutionary associations within a gene family. The 181 *R2R3-SsMYB* genes were found to have varying numbers of exons, that ranged from 1 to 11. Most of the *R2R3-SsMYBs*, like those in other plant species, had three exons and two introns (Jiang et al., 2004; Liu et al., 2014). In this research, the exon/intron patterns of *R2R3-SsMYB* genes exhibited similarity within the same subfamily, with the highest number of introns not exceeding two in most of the genes. These data were congruent with prior research that exhibited the presence of two introns (at maximum) in most land plant *R2R3-MYB* genes (Yin et al., 2022). The majority of *R2R3-MYB* genes belonging to the same subgroup had similar functions and were likely to exhibit similar motif compositions, but there were significant differences among subgroups. For example, subgroup A4 contained motifs 5 and 8, which are involved in inhibiting anthocyanin synthesis, while subgroup A6 contained motifs 7 and 9, which help to promote anthocyanin synthesis (Liu et al., 2021). Therefore, these motifs were conserved within specific subgroups, and proteins in the same subgroup that share these motifs likely have similar functions.

### Gene duplication of *R2R3-MYB* genes in *Spatholobus suberectus*

Gene duplication is a major factor involved in the expansion of gene families and the generation of new genes. The form of gene replication in plants includes whole-genome duplication (WGD) events, and tandem as well as segmental duplication (Zheng et al., 2015). Based on our previous study, two WGD events in *S. suberectus*, and three in *G. max* were identified (Qin et al., 2020), which might result in a significantly lower number of *R2R3-SsMYB* (181) than *R2R3-GmMYB* (244). Gene duplication, when it occurs in different chromosomes, can be termed segmental duplication. However, within the same chromosomes, this is termed tandem duplication. These duplication types are the driving factor behind the diversity of species, which in turn might be crucial for enabling the plants to adapt to continuously changing environments (Qiao et al., 2019; Schilling et al., 2020). The current research documented that the expansion of *R2R3-SsMYB* could be linked to both the aforementioned duplication events, which bear similarity to those in *Nicotiana tabacum* (Yang et al., 2022). There were 99 segmental duplications and one tandem duplication for *R2R3-MYB* genes in *S. suberectus*, implying that the former events were a primary cause of the expansion of *R2R3-SsMYB* genes. Ka/Ks analysis implied that most of the *R2R3-SsMYB* genes were subjected to purifying selection, indicating high conservation during evolution. Nonetheless, some *R2R3-SsMYB* genes were found to be under positive selection, implying that they may have acquired new functions during evolution. Comparative orthologous analysis showed that there was a large amount of collinearity between *S. suberectus* and *A. thaliana*, and between *S. suberectus* and *G. max*, indicating the presence of gene duplication at the level of chromosomes.

### Candidate *R2R3-SsMYB* genes significantly associated with flavonoid synthesis

Prior research has implicated *R2R3-MYB* genes in essential functions in the flavonoid biosynthesis in a variety of plants, such as *EbMYBP1* as a regulator implicated in the regulation of flavonoid accumulation in *Erigeron breviscapus* (Zhao et al., 2022). In *A. thaliana*, *AtMYB44*, *AtMYB123*, and *AtMYB112* have exhibited involvement in the modulation of flavonoid biosynthesis (Lepiniec et al., 2006; Jung et al., 2010; Lotkowska et al., 2015). In this study, 22 candidate *R2R3-SsMYB* genes were significantly associated with flavonoid synthesis, with *SsMYB26*, *SsMYB126*, and *SsMYB* 133 clustered into the same group as *AtMYB44*, *AtMYB123*, and *AtMYB112*, indicating that these genes might be participating in the synthesis of flavonoid. These genes should be assessed in-depth regarding any possible functions they execute through further research.

## Conclusion

This research provided a thorough genome-wide analysis of the *R2R3-SsMYB* family. In total, 181 *R2R3-SsMYBs* were determined in *S. suberectus* and categorized into 35 subgroups, among which 174 *R2R3-SsMYB* genes were mapped to 9 chromosomes. The same subgroup of *R2R3-SsMYB* genes displayed conserved motif compositions and similarity in exon-intron structures, reinforcing the outcomes of the phylogenetic analysis. The expansion of the *R2R3-SsMYB* gene family was primarily driven by segmental duplication events, as indicated by the synteny analysis. The Ka/Ks analysis suggested that the *R2R3-SsMYB* gene family underwent purifying selection. In total, 22 *R2R3-SsMYB* genes were remarkably linked to the amount of flavonoid, catechin, genistein, isoliquiritigenin, and formononetin. These results provide insights into the roles of *R2R3-SsMYB* TFs in flavonoid biosynthesis and a foundation for further research characterizing the functions of *R2R3-MYB* genes in *S. suberectus*.

## Data availability statement

The original contributions presented in the study are included in the article/**Supplementary Material**. Further inquiries can be directed to the corresponding authors.

## Author contributions

SQ analyzed data and wrote the paper. SQ and KW designed the project. YL and QL performed experiments. FW, DT and JM helped with the data analysis and examined the results. All authors contributed to the article and approved the submitted version.

## References

[B1] Anwar M. Yu W. Yao H. Zhou P. Allan A. C. Zeng L. (2019). *NtMYB3*, an R2R3-MYB from Narcissus, regulates flavonoid biosynthesis. Int. J. Mol. Sci. 20 (21), 5456. doi: 10.3390/ijms20215456 31683873 PMC6862390

[B2] Aoyagi L. N. Lopes-Caitar V. S. de Carvalho M. Darben L. M. Polizel-Podanosqui A. Kuwahara M. K.. (2014). Genomic and transcriptomic characterization of the transcription factor family R2R3-MYB in soybean and its involvement in the resistance responses to *Phakopsora pachyrhizi* . Plant Sci. 229, 32–42. doi: 10.1016/j.plantsci.2014.08.005 25443831

[B3] Bailey T. L. Boden M. Buske F. A. Frith M. Grant C. E. Clementi L.. (2009). MEME SUITE: tools for motif discovery and searching. Nucleic Acids Res. 37 (Web Server issue), W202–W208. doi: 10.1093/nar/gkp335 19458158 PMC2703892

[B4] Chao J. Li Z. Sun Y. Aluko O. O. Wu X. Wang Q.. (2021). MG2C: a user-friendly online tool for drawing genetic maps. Mol. Hortic. 1, 16. doi: 10.1186/s43897-021-00020-x PMC10514940 37789491

[B5] Chen C. Chen H. Zhang Y. Thomas H. R. Frank M. H. He Y.. (2020). TBtools: an integrative toolkit developed for interactive analyses of big biological data. Mol. Plant 13 (8), 1194–1202. doi: 10.1016/j.molp.2020.06.009 32585190

[B6] Chen G. He W. Guo X. Pan J. (2021). Genome-wide identification, classification and expression analysis of the MYB transcription factor family in Petunia. Int. J. Mol. Sci. 22 (9), 4838. doi: 10.3390/ijms22094838 34063617 PMC8124715

[B7] Du H. Yang S. S. Liang Z. Feng B. R. Liu L. Huang Y. B.. (2012). Genome-wide analysis of the MYB transcription factor superfamily in soybean. BMC Plant Biol. 12, 106. doi: 10.1186/1471-2229-12-106 22776508 PMC3462118

[B8] Dubos C. Stracke R. Grotewold E. Weisshaar B. Martin C. Lepiniec L. (2010). MYB transcription factors in Arabidopsis. Trends Plant Sci. 15 (10), 573–581. doi: 10.1016/j.tplants.2010.06.005 20674465

[B9] Ferradas Y. Rey L. Martinez O. Rey M. Gonzalez M. V. (2016). Identification and validation of reference genes for accurate norMalization of real-time quantitative PCR data in kiwifruit. Plant Physiol. Biochem. 102, 27–36. doi: 10.1016/j.plaphy.2016.02.011 26897117

[B10] Finn R. D. Clements J. Eddy S. R. (2011). HMMER web server: interactive sequence similarity searching. Nucleic Acids Res. 39 (Web Server issue), W29–W37. doi: 10.1093/nar/gkr367 21593126 PMC3125773

[B11] Finn R. D. Coggill P. Eberhardt R. Y. Eddy S. R. Mistry J. Mitchell A. L.. (2016). The Pfam protein families database: towards a more sustainable future. Nucleic Acids Res. 44 (D1), D279–D285. doi: 10.1093/nar/gkv1344 26673716 PMC4702930

[B12] Huang W. Lv H. Wang Y. (2017). Functional characterization of a novel R2R3-MYB transcription factor modulating the flavonoid biosynthetic pathway from *Epimedium sagittatum* . Front. Plant Sci. 8. doi: 10.3389/fpls.2017.01274 PMC5515856 28769969

[B13] Ito M. (2005). Conservation and diversification of three-repeat Myb transcription factors in plants. J. Plant Res. 118 (1), 61–69. doi: 10.1007/s10265-005-0192-8 15703854

[B14] Jiang C. Gu X. Peterson T. (2004). Identification of conserved gene structures and carboxy-terminal motifs in the Myb gene family of *Arabidopsis* and *Oryza sativa* L. ssp. *indica* . Genome Biol. 5 (7), R46. doi: 10.1186/gb-2004-5-7-r46 15239831 PMC463303

[B15] Jung C. Shim J. S. Seo J. S. Lee H. Y. Kim C. H. Choi Y. D.. (2010). Non-specific phytohormonal induction of *AtMYB44* and suppression of jasmonate-responsive gene activation in *Arabidopsis thaliana* . Mol. Cells 29 (1), 71–76. doi: 10.1007/s10059-010-0009-z 20016937

[B16] Katiyar A. Smita S. Lenka S. K. Rajwanshi R. Chinnusamy V. Bansal K. C. (2012). Genome-wide classification and expression analysis of MYB transcription factor families in rice and *Arabidopsis* . BMC Genomics 13, 544. doi: 10.1186/1471-2164-13-544 23050870 PMC3542171

[B17] Krzywinski M. Schein J. Birol I. Connors J. Gascoyne R. Horsman D.. (2009). Circos: an information aesthetic for comparative genomics. Genome Res. 19 (9), 1639–1645. doi: 10.1101/gr.092759.109 19541911 PMC2752132

[B18] Lepiniec L. Debeaujon I. Routaboul J. M. Baudry A. Pourcel L. Nesi N.. (2006). Genetics and biochemistry of seed flavonoids. Annu. Rev. Plant Biol. 57, 405–430. doi: 10.1146/annurev.arplant.57.032905.105252 16669768

[B19] Liu J. Wang J. Wang M. Zhao J. Zheng Y. Zhang T.. (2021). Genome-wide analysis of the R2R3-MYB gene family in *Fragaria* × *ananassa* and its function identification during anthocyanins biosynthesis in pink-flowered strawberry. Front. Plant Sci. 12. doi: 10.3389/fpls.2021.702160 PMC8435842 34527006

[B20] Liu C. Wang X. Xu Y. Deng X. Xu Q. (2014). Genome-wide analysis of the R2R3-MYB transcription factor gene family in sweet orange (*Citrus sinensis*). Mol. Biol. Rep. 41 (10), 6769–6785. doi: 10.1007/s11033-014-3563-1 25008995

[B21] Lotkowska M. E. Tohge T. Fernie A. R. Xue G. P. Balazadeh S. Mueller-Roeber B. (2015). The *Arabidopsis* transcription factor *MYB112* promotes anthocyanin formation during salinity and under high light stress. Plant Physiol. 169 (3), 1862–1880. doi: 10.1104/pp.15.00605 26378103 PMC4634054

[B22] Ogata K. Kanei-Ishii C. Sasaki M. Hatanaka H. Nagadoi A. Enari M.. (1996). The cavity in the hydrophobic core of Myb DNA-binding domain is reserved for DNA recognition and trans-activation. Nat. Struct. Biol. 3 (2), 178–187. doi: 10.1038/nsb0296-178 8564545

[B23] Peng F. Meng C. W. Zhou Q. M. Chen J. P. Xiong L. (2016). Cytotoxic evaluation against breast cancer cells of isoliquiritigenin analogues from *Spatholobus suberectus* and their synthetic derivatives. J. Nat. Prod. 79 (1), 248–251. doi: 10.1021/acs.jnatprod.5b00774 26690274

[B24] Price M. N. Dehal P. S. Arkin A. P. (2009). FastTree: computing large minimum evolution trees with profiles instead of a distance matrix. Mol. Biol. Evol. 26 (7), 1641–1650. doi: 10.1093/molbev/msp077 19377059 PMC2693737

[B25] Pu X. Yang L. Liu L. Dong X. Chen S. Chen Z.. (2020). Genome-wide analysis of the MYB transcription factor superfamily in *Physcomitrella patens* . Int. J. Mol. Sci. 21 (3), 975. doi: 10.3390/ijms21030975 32024128 PMC7037163

[B26] Pucker B. Pandey A. Weisshaar B. Stracke R. (2020). ). The R2R3-MYB gene family in banana (*Musa acuminata*): genome-wide identification, classification and expression patterns. PloS One 15, e0239275. doi: 10.1371/journal.pone.0239275 33021974 PMC7537896

[B27] Qiao X. Li Q. Yin H. Qi K. Li L. Wang R.. (2019). Gene duplication and evolution in recurring polyploidization-diploidization cycles in plants. Genome Biol. 20 (1), 38. doi: 10.1186/s13059-019-1650-2 30791939 PMC6383267

[B28] Qin S. Wei K. Cui Z. Liang Y. Li M. Gu L.. (2020). Comparative genomics of *Spatholobus suberectus* and insight into flavonoid biosynthesis. Front. Plant Sci. 11. doi: 10.3389/fpls.2020.528108 PMC7500164 33013959

[B29] Qin S. Wu L. Wei K. Liang Y. Song Z. Zhou X.. (2019). A draft genome for *Spatholobus suberectus* . Sci. Data 6 (1), 113. doi: 10.1038/s41597-019-0110-x 31273216 PMC6609623

[B30] Riechmann J. L. Heard J. Martin G. Reuber L. Jiang C. Keddie J.. (2000). *Arabidopsis* transcription factors: genome-wide comparative analysis among eukaryotes. Science 290 (5499), 2105–2110. doi: 10.1126/science.290.5499.2105 11118137

[B31] Schilling S. Kennedy A. Pan S. Jermiin L. S. Melzer R. (2020). Genome-wide analysis of MIKC-type MADS-box genes in wheat: pervasive duplications, functional conservation and putative neofunctionalization. New Phytol. 225 (1), 511–529. doi: 10.1111/nph.16122 31418861

[B32] Schmutz J. Cannon S. Schlueter J. Ma J. Mirtos T. Nelson W.. (2010). Genome sequence of the palaeopolyploid soybean. Nature 463, 178–183. doi: 10.1038/nature08670 20075913

[B33] Song H. K. Park S. H. Kim H. J. Jang S. Kim T. (2022). *Spatholobus suberectus* Dunn water extract ameliorates atopic dermatitis-like symptoms by suppressing proinflammatory chemokine production in *vivo* and in *vitro* . Front. Pharmacol. 13. doi: 10.3389/fphar.2022.919230 PMC9251377 35795574

[B34] Sundararaman G. Sadasivam B. Gajjeraman P. (2015). The MYB transcription factor family genes in Sugarcane (*Saccharum* sp.). Plant Mol. Biol. Rep. 33 (3), 512–531. doi: 10.1007/s11105-014-0768-3

[B35] The Arabidopsis Genome Initiative (2000). Analysis of the genome sequence of the flowering plant *Arabidopsis thaliana* . Nature 408, 796–815. doi: 10.1038/35048692 11130711

[B36] Wang H. Liu Y. N. Zeng Z. P. He W. (2011). Study on HPLC chromatographic fingerprint of anti-tumor active site SSCE of Caulis spatholobi. Zhongguo Zhongyao Zazhi 36, 2525–2529. doi: 10.4268/cjcmm20111816 22256759

[B37] Wang X. Li Y. Liu Y. Zhang D. Ni M. Jia B.. (2021). Transcriptomic and proteomic profiling reveal the key role of *AcMYB16* in the response of *Pseudomonas syringae* pv. *actinidiae* in Kiwifruit. Front. Plant Sci. 12. doi: 10.3389/fpls.2021.756330 PMC8632638 34868148

[B38] Wang D. Liu P. Chen Y. Chen R. Guo D. Ren H.. (2008). Stimulating effect of catechin, an active component of *Spatholobus suberectus* Dunn, on bioactivity of hematopoietic growth factor. Chin. Med. J. 121 (8), 752–755. doi: 10.1186/1471-2296-9-22 18701032

[B39] Wang Y. Tang H. Debarry J. D. Tan X. Li J. Wang X.. (2012). MCScanX: a toolkit for detection and evolutionary analysis of gene synteny and collinearity. Nucleic Acids Res. 40 (7), e49. doi: 10.1093/nar/gkr1293 22217600 PMC3326336

[B40] Wang D. Zhang Y. Zhang Z. Zhu J. Yu J. (2010). KaKs_Calculator 2.0: a toolkit incorporating gamma-series methods and sliding window strategies. Genomics Proteomics Bioinf. 8 (1), 77–80. doi: 10.1016/S1672-0229(10)60008-3 PMC5054116 20451164

[B41] Wang L. X. Zheng H. R. Ren F. C. Chen T. G. Li X. M. Jiang X. J.. (2017). Polysubstituted isoflavonoids from *Spatholobus suberectus*, *Flemingia macrophylla*, and *Cudrania cochinchinensis* . Nat. Prod. Bioprospect 7 (2), 201–206. doi: 10.1007/s13659-017-0121-2 28110438 PMC5397389

[B42] Yang J. Zhang B. Gu G. Yuan J. Shen S. Jin L.. (2022). Genome-wide identification and expression analysis of the R2R3-MYB gene family in tobacco (*Nicotiana tabacum* L.). BMC Genomics 23 (1), 432. doi: 10.1186/s12864-022-08658-7 35681121 PMC9178890

[B43] Yin Y. Guo C. Shi H. Zhao J. Ma F. An W.. (2022). Genome-wide comparative analysis of the *R2R3-MYB* gene family in five Solanaceae species and identification of members regulating carotenoid biosynthesis in Wolfberry. Int. J. Mol. Sci. 23 (4), 2259. doi: 10.3390/ijms23042259 35216373 PMC8875911

[B44] Yuan Y. Yang X. Feng M. Ding H. Khan M. T. Zhang J.. (2021). Global dissection of R2R3-MYB in *Pogostemon cablin* uncovers a species-specific R2R3-MYB clade. BMC Genomics 22 (1), 622. doi: 10.1186/s12864-021-07689-w 37217084

[B45] Zeng Y. Li Z. Chen Y. Li W. Wang H. B. Shen Y. (2023). Characterization of wheat MYB genes responsive to high temperatures. Genomics 115 (4), 110643. doi: 10.1016/j.ygeno.2023.110643 37217084

[B46] Zhao Y. Tian X. Wang F. Zhang L. Xin M. Hu Z.. (2017). Characterization of wheat MYB genes responsive to high temperatures. BMC Plant Biol. 17 (1), 208. doi: 10.1186/s12870-017-1158-4 29157199 PMC5696766

[B47] Zhao Y. Zhang G. Tang Q. Song W. Gao Q. Xiang G.. (2022). *EbMYBP1*, a R2R3-MYB transcription factor, promotes flavonoid biosynthesis in *Erigeron breviscapus* . Front. Plant Sci. 13. doi: 10.3389/fpls.2022.946827 PMC9366350 35968130

[B48] Zheng C. Santos M. D. Albert V. A. Sankoff D. (2015). Syntenic block overlap multiplicities with a panel of reference genomes provide a signature of ancient polyploidization events. BMC Genomics 16 (Suppl 10), S8. doi: 10.1186/1471-2164-16-S10-S8 PMC4603330 26449933

[B49] Zhuang W. Shu X. Lu X. Wang T. Zhang F. Wang N.. (2021). Genome-wide analysis and expression profiles of *PdeMYB* transcription factors in colored-leaf poplar (*Populus deltoids*). BMC Plant Biol. 21 (1), 432. doi: 10.1186/s12870-021-03212-1 34556053 PMC8459500

